# Supplementary figures and images for: Asymmetrical interference between number and item size perception provides evidence for a domain specific impairment in dyscalculia
Source: PLoS One. 2018 Dec 14;13(12):e0209256. doi: 10.1371/journal.pone.0209256 (PMC6294370; doi:10.1371/journal.pone.0209256)

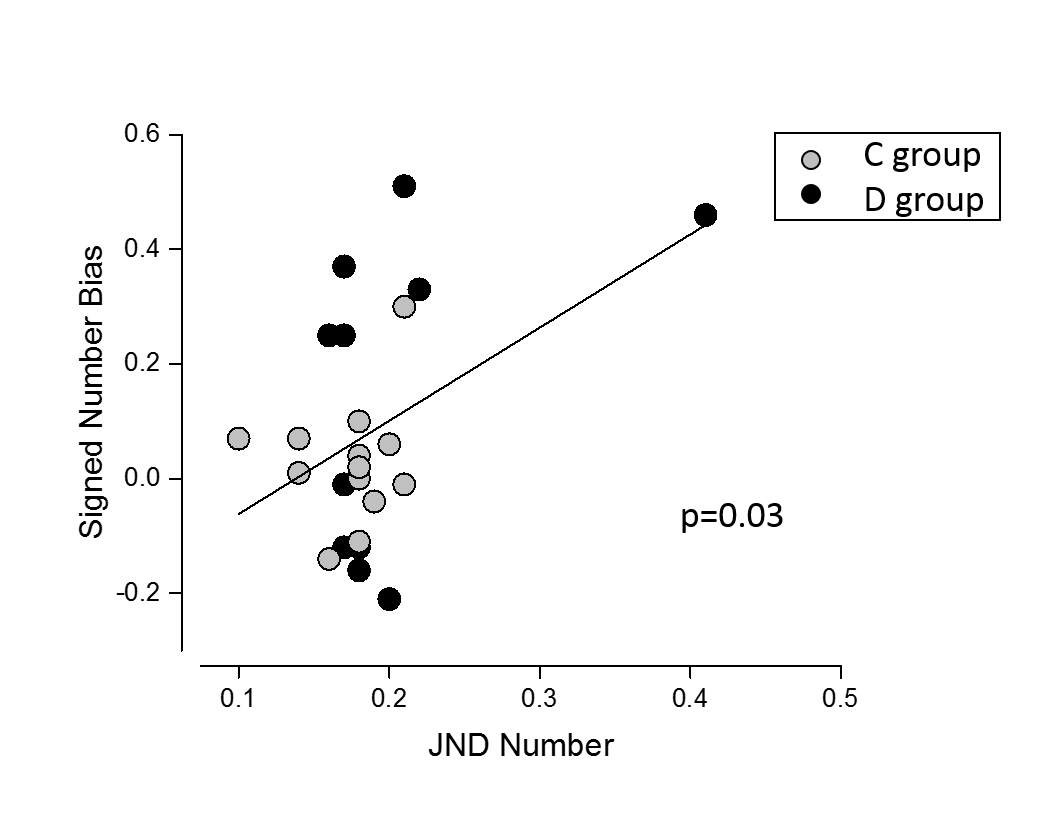

Supplement: S1 Fig — Correlation between the signed PSE number bias and the overall precision during and numerosity judgments. Gray and black circles represents participants of the control and dyscalculic group, respectively. (TIF) [file pone.0209256.s002.tif]
